# Introducing a new auto edge detection technique capable of revealing cervical root resorption in CBCT scans with pronounced metallic artifacts

**DOI:** 10.1038/s41598-024-54974-1

**Published:** 2024-02-20

**Authors:** Negar Khosravifard, Bardia Vadiati Saberi, Amir Khosravifard, Amirreza Hendi, Kimia Shadi, Sanaz Mihandoust, Zahra Yousefi, Tahereh Mortezaei, Mohammad Ebrahim Ghaffari

**Affiliations:** 1 Department of Oral and Maxillofacial Radiology, Dental Sciences Research Center, School of Dentistry, Guilan University of Medical Sciences, Rasht, Iran; 2 Department of Periodontics, Dental Sciences Research Center, School of Dentistry, Guilan University of Medical Sciences, Rasht, Iran; 3 Department of Mechanical Engineering, Shiraz University, Shiraz, Iran; 4 Department of Dental Prosthesis, Dental Sciences Research Center, School of Dentistry, Guilan University of Medical Sciences, Rasht, Iran; 5 Department of Oral and Maxillofacial Radiology, Dental Caries Prevention Research Center, School of Dentistry, Qazvin University of Medical Sciences, Qazvin, Iran; 6 Department of Biostatistics and Epidemiology, QomUniversity of Medical Sciences, Qom, Iran

**Keywords:** Image processing, Computer-assisted, Endodontics, Cone-beam computed tomography, Artifacts, Dental conditions, Dental radiology, Endodontics

## Abstract

Cervical resorption is a serious threat to the longevity of the teeth. In this study, the Canny edge-detection algorithm was applied on CBCT images to compare the accuracy of original and Canny views for diagnosing cervical resorption in endodontically treated teeth. Intracanal metallic posts were inserted in 60 extracted teeth being randomly divided into three groups: control, 0.5 mm, and 1 mm cervical resorption. CBCT scans of the teeth were presented to three observers in both original and Canny formats with the accuracy being determined by receiver operating characteristic (ROC) analysis. The DeLong test was used for paired comparisons with the significance level set at 0.05. The highest accuracy belonged to Canny images in 1 mm resorption, followed by Canny images in 0.5 mm resorption, original images in 1 mm resorption, and original images in 0.5 mm resorption, respectively. The Canny images were significantly more accurate in the diagnosis of 0.5 mm (*p* < 0.001) and 1 mm (*p* = 0.009) resorption. Application of the Canny edge-detection algorithm could be suggested as a new technique for facilitating the diagnosis of cervical resorption in teeth that are negatively affected by metallic artifacts.

## Introduction

External cervical resorption (ECR) is defined as an irreversible loss of dental structures (i.e., cementum and dentin) followed by the action of odontoclastic cells^1^. Such resorption resulting from mechanical or chemical factors can be associated with orthodontic treatment, ectopic eruption, tooth impaction, parafunctional occlusion, periodontal/periapical infections, restorations, internal bleaching, trauma, and some systemic conditions such as Paget’s disease^1–6^. The process of resorption initiates at some point below the epithelial attachment and can gradually progress into the entire cervical region^7^. ECR is mainly asymptomatic and would incidentally be detected through radiographic examinations unless it involves the pulp or periodontal tissues. Since effective treatments for ECR are needed to be timely, precise identification of their location and magnitude is crucial^4,8–10^.

Conventional radiographs are routinely used as the first modality for ECR detection^10^. However, because of the limitations of two-dimensional radiographs such as superimposition of the anatomical structures, uneven magnification, and distortion; panoramic and periapical radiographs could result in misdiagnosis by having false-positive and false-negative results^4,10–12^. Cone beam computed tomography (CBCT) has proved to be an efficient method for diagnosis of ECR^12,13^. According to the previous studies, CBCT scans using a 0.3 mm voxel size would demonstrate root resorption with high accuracy^14^. Nevertheless, streaking artifacts generated from high-density materials such as intracanal posts and metallic restorations could corrupt the CBCT data by disfiguring the anatomical structures^15–23^. Such artifacts could jeopardize the diagnosis of lesions such as ECR especially when they are small and therefore postpone their treatment. Hence, developing techniques for enhancing the visibility of ECR lesions in the presence of metallic artifacts is necessary.

Canny edge-detection algorithm has been introduced as a technique for identifying the outlines of an object as well as sharp intensity changes in the images^24,25^. It has the capability of detecting the outer borders of an object precisely, without being affected by exposure parameters or external conditions^17^. This algorithm has been applied for quantification of metal artifacts in CBCT, diagnosis of osteoporosis, and detection of dental implant fractures^15,17,26^. Nevertheless, no studies so far have used the Canny algorithm to determine whether it facilitates the diagnosis of cervical resorption in the presence of metallic artifacts. Following the STARD guidelines^27^, for the first time we assessed the performance of this algorithm in the diagnosis of ECR defects when metal artifacts generated from intracanal posts worsens the condition.

## Methods

### Study design and sample preparation

This experimental in vitro study was approved by the Research Ethics Committee of Guilan University of Medical Sciences (Approval ID: IR.GUMS.REC.1401.039). All experiments were performed in accordance with relevant guidelines and regulations of the Declaration of Helsinki.

**Figure 1:**
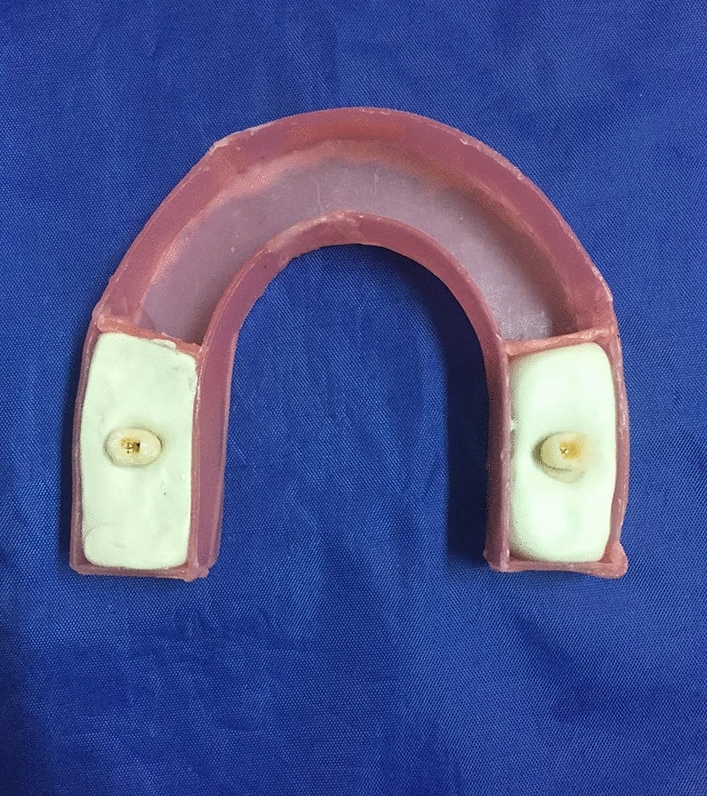
Wax arch containing a pair of teeth for each CBCT exposure.

60 human premolar teeth that were extracted for orthodontic reasons were selected. Extraction of the teeth was performed as part of the patients’ orthodontic treatments and informed consent was obtained from all subjects to use their extracted teeth as the samples of the present study. All selected teeth had a single root. Teeth with caries, restorations, or anatomical concavities in the cervical region as well as any evidence of root resorption or crack were excluded. Root canal treatment was performed for all of the premolar teeth and the canals were obturated with gutta-percha points (Meta Biomed; Cheongju-si, Korea). Afterwards, post spaces with 8 mm length were prepared using Peeso reamers no. 2, 3, and 4. A prefabricated size 2 titanium post (Nordin; Montreux, Switzerland) was passively inserted in the root canals and self-cured luting glass ionomer cement (Fuji I; GC, Tokyo, Japan) was applied. The teeth were then divided into 3 groups randomly: 1- teeth with no cervical resorption (control group), 2- teeth with 0.5 mm cervical resorption, and 3- teeth with 1 mm cervical resorption. The resorption cavities were created at the CEJ of the teeth with two different depths (0.5 and 1 mm) using a round diamond bur. Following that, the margins were beveled with a flame diamond bur to create more natural shapes for the resorption defects. Depth of the cavities was controlled and measured with the use of a periodontal probe. In order to simulate the shape and density of the surrounding soft tissues, each tooth was inserted in a condensational type impression material (Speedex; Coltene, Altstatten, Switzerland) which was placed within a wax arch formed in the shape of the mandible (Fig. 1).

### Radiographic examinations and image processing

**Figure 2:**
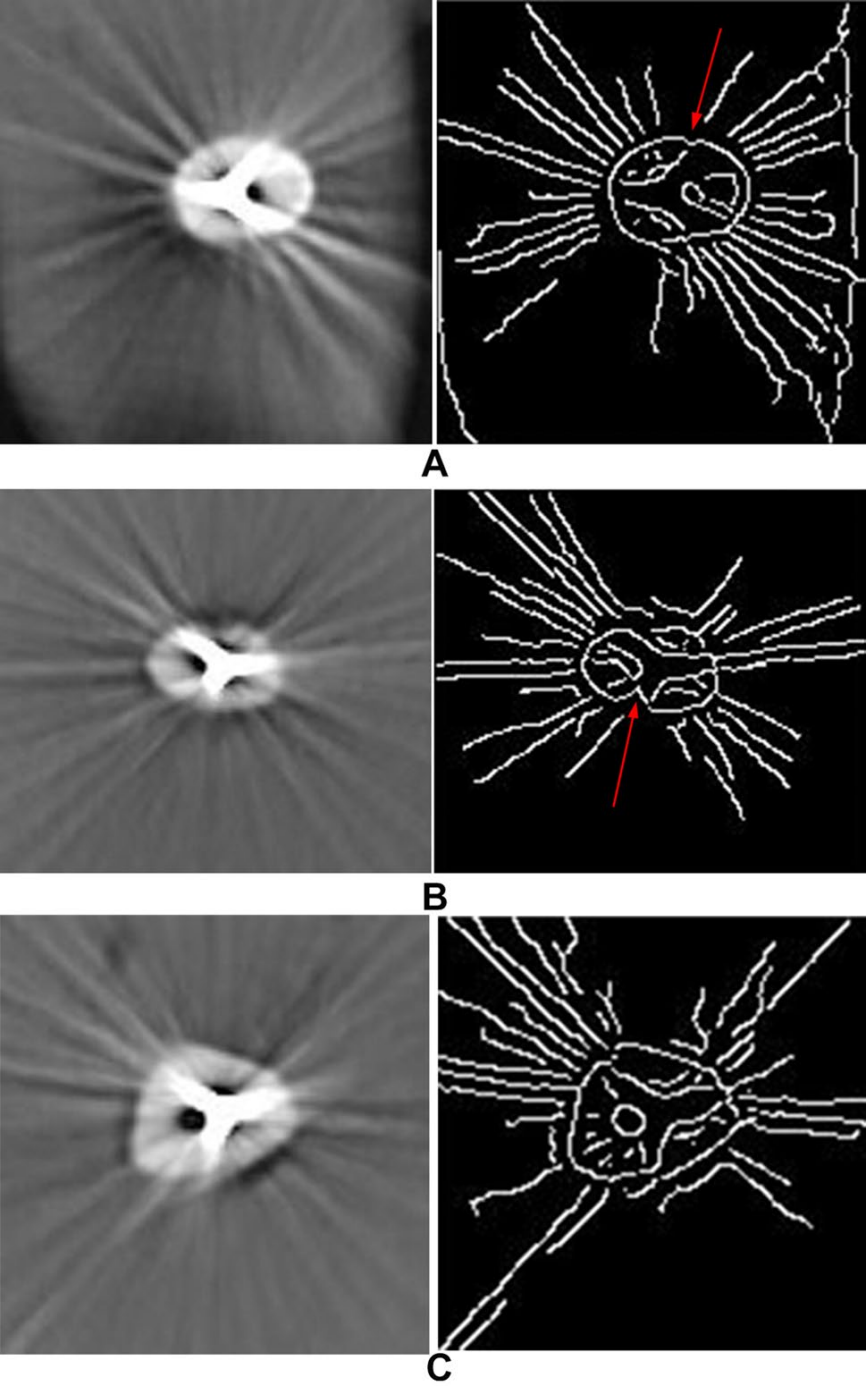
Comparison of the original (left) and Canny (right) images in displaying the resorption defects of 0.5 mm (**A**), 1 mm (**B**), and control group (**C**). Arrows on the Canny images point to the resorption areas that are unidentifiable on the original images.

The wax arch contained 2 teeth that were randomly selected for each scanning procedure. The arch was placed in the central position of a CBCT unit (Pax-i 3D; Vatech, Hwaseong-si, Korea) and exposure parameters included 95 kV, 5.2 mA, FOV of 120 mm × 90 mm, and voxel size of 0.2 mm. Ez3D-i software version 1.1 (Ez3D-i; Vatech, Hwaseong-si, Korea, Available at: https://www.vatech.com/software_3d/417) was used to reconstruct the CBCT data. Three successive axial slices that referred to the cervical region of each tooth were selected. The selected images were saved in their original formats and at the same time exported to MATLAB version 2018a (MathWorks; Natick, MA, USA) to create the Canny-type images. In this way, 6 images including 3 original and 3 canny types were prepared from the CBCT scan of each tooth. In applying the Canny edge detection algorithm, firstly, image noise is removed by a Gaussian filter. Then, using a gradient magnitude thresholding technique, the edges of the image are identified. These steps are sequentially performed by MATLAB version 2018a software (MathWorks; Natick, MA, USA) in an automatic manner. Three parameters including standard deviation of the Gaussian filter, high, and low thresholds are defined by the operator. In the current study, standard deviation of the Gaussian filter was set to 1.2 and high and low thresholds were selected at 0.07 and 0.028, respectively^17^. All images were randomly numbered to be presented to the observers. Random numbering of the original and Canny images was performed to ensure no bias occurs during evaluation of the images. The observers included 3 experienced oral and maxillofacial radiologists who were asked to determine whether ECR existed in the images or not. Figure 2 refers to the original and Canny-type images of two teeth with different depths of cervical resorption.

### Analysis

Diagnostic accuracy of the index tests (original and Canny images) against the reference standard (direct observation of the teeth) is reported following the STARD guidelines^27^. Calculation of the index tests sensitivity and specificity as well as receiver operating characteristic (ROC) analysis was performed by the MedCalc software version 20.026 (MedCalc; Ostend, Belguim). The standard error (SE) was calculated with the DeLong test and confidence interval (CI) for the area under the ROC curve (AUC) was defined by the Binominal exact method. Pairwise comparisons were also performed using the DeLong test. *P* values less than 0.05 were considered to be statistically significant. The inter-rater reliability was defined by kappa coefficient which was over 0.7 between each pair of the three observers. The kappa values were measured using SPSS software version 28 (IBM; Armonk, NY, USA).

## Results

**Figure 3:**
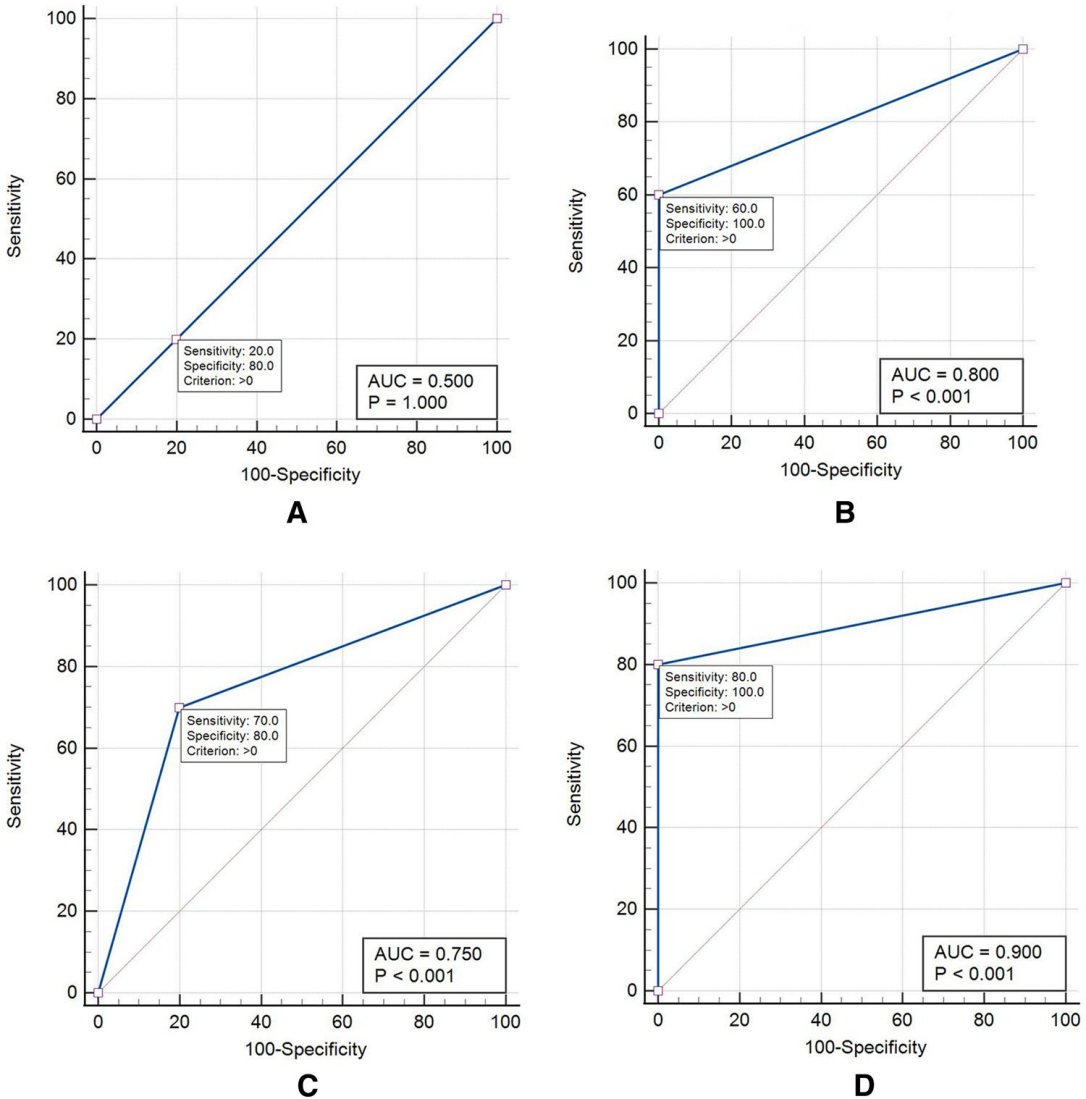
ROC curves of the original and Canny images in the different resorption depths: (**A**) original images in 0.5 mm depth, (**B**) Canny images in 0.5 mm depth, (**C**) original images in 1 mm depth, (**D**) Canny images in 1 mm depth.

Table 1 presents the sensitivity, specificity, and AUC values for the original and Canny images in detecting different depths of cervical resorption. It is noteworthy to mention that the significance values in Table 1 refer to the comparison of each image type with the random technique which is a technique with 50% chance of correct diagnosis. *P* values in Table 1 suggest that both image types are significantly more accurate than the random technique except for the original images in detecting 0.5 mm resorption cavities. ROC curves for the different image types are presented in Fig. 3.

**Table 1:** Diagnostic accuracy of the original and Canny images for the detection of ECR.

Image type	Resorption depth (mm)	AUC	SE^a^	95% CI^b^	*p* value	Sensitivity	Specificity
Original	0.5	0.5	0.065	0.34–0.66	> 0.999	0.20	0.80
	1	0.75	0.070	0.59–0.87	< 0.001	0.70	0.80
Canny	0.5	0.8	0.056	0.64–0.91	< 0.001	0.60	1.00
	1	0.9	0.046	0.76–0.97	< 0.001	0.80	1.00

AUC area under the ROC curve.

*SE* standard error, *CI* confidence interval.

^a^DeLong test.

^b^Binominal exact test.

Comparison of the original and Canny images for the diagnosis of different depths of cervical resorption showed that in both 0.5 and 1 mm cavities, Canny images were superior to the original ones in terms of revealing the resorption defects. Table 2 refers to the comparison of the two image types in identifying the resorption cavities in the teeth.

**Table 2:** Comparison of the diagnostic accuracy of original and Canny images in detecting ECR.

Resorption depth (mm)	Difference in AUC	SE^a^	95% CI^b^	Test statistics	*p* value
0.5	0.30	0.072	0.16–0.44	4.13	< 0.001
1	0.15	0.057	0.04–0.26	2.61	0.009

*SE* standard error, *CI* confidence interval.

^a^DeLong test.

^b^Binominal exact test.

**Figure 4:**
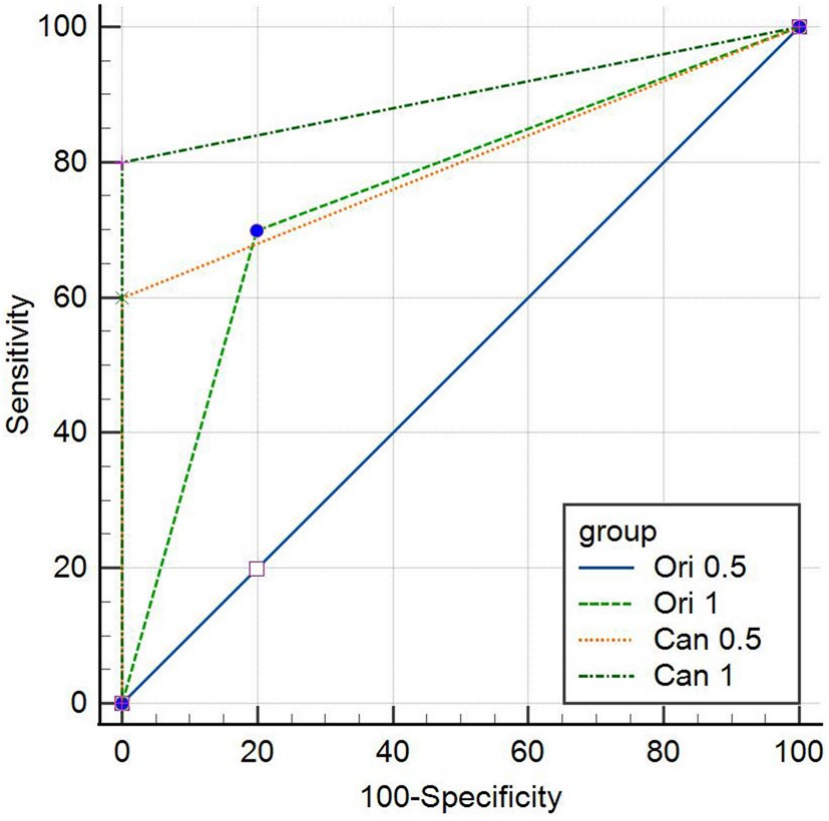
Comparison of AUC among the original and Canny images in 0.5 mm and 1 mm resorption cavities.

Considering the depth of resorption cavities, accuracy of the original images was significantly different between the two resorption depths (*p* = 0.009). The accuracy was higher in the detection of larger cavities which were 1 mm deep. Nevertheless, no significant difference existed in the diagnosis of 0.5 and 1 mm cavities by the Canny images (*p* = 0.168). Figure 4 graphically shows the comparison of AUC values among different image types and resorption depths. The highest accuracy belonged to Canny images for 1 mm defects, followed by Canny images for 0.5 mm defects, original images for 1 mm defects, and original images for 0.5 mm defects, respectively.

## Discussion

Root resorption results in loss of dental hard tissue leading to reduction of crown-root ratio or loss of the whole tooth in severe cases^28,29^. ECR usually initiates more apically than the epithelial attachment and can progress in any direction^7^. This pathologic condition occurs in response to trauma, failure of periodontal or root canal treatments, undesirable position of the nearby impacted teeth, and ectopic eruption^2–4^. Since ECR is usually asymptomatic, better prognosis can be expected with an early diagnosis^30^. Having more accuracy than 2D plain radiographies, CBCT images are more reliably used for the diagnosis of root resorption^1,31,32^. Moreover, the influence of parameters such as voxel size and FOV dimensions on the diagnostic accuracy of root resorption has been investigated previously^33–36^. Nevertheless, treated teeth with root canal filling material and intracanal metallic posts produce interfering artifacts that make the diagnosis of root resorption difficult^31^. A number of previous studies have assessed the influence of artifacts caused from endodontically treated teeth on the accuracy of CBCT images^10,17,37^; however, no studies so far have investigated the accuracy of CBCT images for the diagnosis of ECR when intracanal metallic posts are present.

In the present study, CBCT images of three tooth groups based on the presence and depth of ECR (0, 0.5, and 1 mm) were examined. We created the resorption defects at the cervical region of the teeth since the intensity of artifacts from metallic posts is greatest at this region. According to the previous studies, an increased cavity size of ECR improves its detection^32,38,39^. Neves et al. concluded that better sensitivity, accuracy, positive and negative predictive values were obtained with larger defects^38,39^. Similarly, in our research, ECR defects with 1 mm depth were more accurately diagnosed than those with 0.5 mm depth. Nikneshan et al.^35^ reported that although increasing the size of defects increases the diagnosis accuracy, the difference is not statistically significant. Lack of a significant difference between the depth groups could be attributed to the absence of root filling material, metal restorations, or intracanal metallic posts and hence, lack of metallic artifacts. Neves et al. reported that an intracanal metallic post significantly decreases the accuracy of CBCT images for the diagnosis of vertical root fractures^39^. Thus, developing techniques that are capable of compensating for the adverse effects of metal artifacts is of utmost importance in diagnostic radiology.

In the present study, we used the Canny edge-detection technique as a novel method to determine whether it could improve the CBCT images in a way that the diagnosis of ECR in the vicinity of metallic posts is facilitated. We compared the detectability of ECR among original and Canny images derived from CBCT scans. The Canny edge-detection technique uses an algorithm which provides an accurate instrument for defining the object's outlines and sudden intensity changes in an image. Three parameters of the Canny algorithm including standard deviation of the Gaussian filter, high and low sensitivity thresholds can be set manually by the operator. Previous studies have suggested certain values for these three parameters to achieve the best image quality^15,17^. In this study, two experienced oral and maxillofacial radiologists who were not among the observers viewed the images to confirm that the suggested values for the aforementioned parameters provided the finest image details.

In order to assess the accuracy of different image types, original and Canny images were first compared with the random technique. Subsequently, the two image types (original and Canny) were compared with each other. The only image type that showed no significant difference with the random technique was original images in 0.5 mm depth of resorption (*p* > 0.999). This means that CBCT images in their original format could not be helpful for the diagnosis of small ECR defects when metallic artifacts exist. In other words, the diagnostic accuracy of CBCT images without using the Canny algorithm is adversely affected by the depth of the defect.

Comparison of the original and Canny images revealed significant differences in the diagnosis of ECR. Canny images were more accurate in either the 0.5 mm (*p* < 0.001) or 1 mm (*p* = 0.009) defect depths. This finding confirms the positive effect of applying the Canny algorithm for the diagnosis of ECR. Another important result that we obtained was that the diagnostic accuracy of Canny images did not differ significantly between the two ECR depths (*p* = 0.168) while the original images performed differently in the depth groups (*p* = 0.009). Hence, it could be concluded that use of the Canny edge-detection technique improves the CBCT images to the point that different ECR sizes are diagnosed with almost similar accuracy.

There are also limitations noteworthy to mention for this in vitro study. First, although resorption cavities were beveled to make them more natural-looking, clinical conditions are frequently associated with more bizarre-shaped defects that make the diagnosis more challenging. Hence, further studies in clinical situations are required to determine the accuracy of Canny edge-detection. Second, different voxel sizes have to be examined with the CBCT scans to assess the accuracy of Canny edge-detection in various resolutions.

## Conclusion

Application of the Canny edge-detection algorithm on CBCT images with interfering metallic artifacts greatly improves the diagnosis of different depths of cervical resorption in endodontically treated teeth.

## Data Availability

The datasets used and/or analyzed during the current study available from the corresponding author on reasonable request.

## References

[1] Schröder ÂG, Westphalen FH, Schröder JC, Fernandes Â, Westphalen VP (2018). Accuracy of digital periapical radiography and cone-beam computed tomography for diagnosis of natural and simulated external root resorption. J. Endod..

[2] Asgary S, Nourzadeh M, Verma P, Hicks ML, Nosrat A (2019). Vital pulp therapy as a conservative approach for management of invasive cervical root resorption: A case series. J. Endod..

[3] Deeb JG, Azarnoush K, Laskin DM, Deeb GR (2019). Discontinuation of denosumab as a potential cause of generalized external cervical root resorption: A case report. J. Endod..

[4] Wang D, He X, Wang Y, Li Z, Zhu Y, Sun C, Ye J, Jiang H, Cheng J (2017). External root resorption of the second molar associated with mesially and horizontally impacted mandibular third molar: Evidence from cone beam computed tomography. Clin. Oral. Investig..

[5] Moshkelgosha V, Khosravifard N, Golkari A (2014). Tooth eruption sequence and dental crowding: A case-control study. F1000Res..

[6] Kamburoğlu K, Kurşun Ş, Yüksel S, Öztaş B (2011). Observer ability to detect ex vivo simulated internal or external cervical root resorption. J. Endod..

[7] Mavridou AM, Hauben E, Wevers M, Schepers E, Bergmans L, Lambrechts P (2016). Understanding external cervical resorption in vital teeth. J. Endod..

[8] Mikušková K, Vaňuga P, Adamicová K, Statelová D, Janíčková M, Malachovský I, Siebert T (2022). Multiple idiopathic external cervical root resorption in patient treated continuously with denosumab: A case report. BMC. Oral. Health..

[9] Oenning AC, Melo SL, Groppo FC, Haiter-Neto F (2015). Mesial inclination of impacted third molars and its propensity to stimulate external root resorption in second molars—A cone-beam computed tomographic evaluation. J. Oral. Maxillofac. Surg..

[10] Dalili Z, Taramsari M, Mehr SZ, Salamat F (2012). Diagnostic value of two modes of cone-beam computed tomography in evaluation of simulated external root resorption: An in vitro study. Imaging. Sci. Dent..

[11] Li D, Tao Y, Cui M, Zhang W, Zhang X, Hu X (2019). External root resorption in maxillary and mandibular second molars associated with impacted third molars: A cone-beam computed tomographic study. Clin. Oral. Investig..

[12] Patel S, Foschi F, Mannocci F, Patel K (2018). External cervical resorption: A three-dimensional classification. Int. Endod. J..

[13] Patel K, Mannocci F, Patel S (2016). The assessment and management of external cervical resorption with periapical radiographs and cone-beam computed tomography: A clinical study. J. Endod..

[14] Liedke GS, da Silveira HE, da Silveira HL, Dutra V, de Figueiredo JA (2009). Influence of voxel size in the diagnostic ability of cone beam tomography to evaluate simulated external root resorption. J. Endod..

[15] Khosravifard N, Saberi BV, Khosravifard A, Zakerjafari H, Vafaei R, Ghaffari ME (2022). A diagnostic accuracy study on an innovative auto-edge detection technique for identifying simulated implant fractures on radiographic images. Sci. Rep..

[16] Schulze RK, Berndt D, d'Hoedt B (2010). On cone-beam computed tomography artifacts induced by titanium implants. Clin. Oral. Implants. Res..

[17] Khosravifard A, Saberi BV, Khosravifard N, Motallebi S, Kajan ZD, Ghaffari ME (2021). Application of an auto-edge counting method for quantification of metal artifacts in CBCT images: A multivariate analysis of object position, field of view size, tube voltage, and metal artifact reduction algorithm. Oral. Surg. Oral. Med. Oral. Pathol. Oral. Radiol..

[18] Freitas DQ, Fontenele RC, Nascimento EH, Vasconcelos TV, Noujeim M (2018). Influence of acquisition parameters on the magnitude of cone beam computed tomography artifacts. Dentomaxillofac. Radiol..

[19] Kajan ZD, Asli HN, Taramsari M, Fallah Chai SM, Babaei Hemmaty Y (2015). Comparison of height and width measurements of mandibular bone in various head orientations using cone beam computed tomography: An experimental in vitro study. Oral. Radiol..

[20] Codari M, de Faria Vasconcelos K, Ferreira Pinheiro Nicolielo L, Haiter Neto F, Jacobs R (2017). Quantitative evaluation of metal artifacts using different CBCT devices, high-density materials and field of views. Clin. Oral. Implants. Res..

[21] Queiroz PM, Santaella GM, da Paz TD, Freitas DQ (2017). Evaluation of a metal artefact reduction tool on different positions of a metal object in the FOV. Dentomaxillofac. Radiol..

[22] Pauwels R, Jacobs R, Bogaerts R, Bosmans H, Panmekiate S (2016). Reduction of scatter-induced image noise in cone beam computed tomography: Effect of field of view size and position. Oral. Surg. Oral. Med. Oral. Pathol. Oral. Radiol..

[23] Queiroz PM, Groppo FC, Oliveira ML, Haiter-Neto F, Freitas DQ (2017). Evaluation of the efficacy of a metal artifact reduction algorithm in different cone beam computed tomography scanning parameters. Oral. Surg. Oral. Med. Oral. Pathol. Oral. Radiol..

[24] Kim YH, Lee C, Han SS, Jeon KJ, Choi YJ, Lee A (2020). Quantitative analysis of metal artifact reduction using the auto-edge counting method in cone-beam computed tomography. Sci. Rep..

[25] Sangeetha D, Deepa P (2019). FPGA implementation of cost-effective robust Canny edge detection algorithm. J. Real-Time. Image. Proc..

[26] Marar RF, Uliyan DM, Al-Sewadi HA (2020). Mandible bone osteoporosis detection using cone-beam computed tomography. Eng. Technol. Appl. Sci. Res..

[27] Bossuyt PM (2015). STARD 2015: An update list of essential items for reporting diagnostic accuracy studies. BMJ..

[28] Patel S, Saberi N, Pimental T, Teng PH (2022). Present status and future directions: Root resorption. Int. Endod. J..

[29] Fang X, Qi R, Liu C (2019). Root resorption in orthodontic treatment with clear aligners: A systematic review and meta-analysis. Orthod. Craniofac. Res..

[30] Aidos H, Diogo P, Santos JM (2018). Root resorption classifications: A narrative review and a clinical aid proposal for routine assessment. Eur. Endod J..

[31] Bastos JV, Queiroz VH, Felício DB, Ferreira DA, Brasileiro CB, Abdo EN, Amaral TM (2020). Imaging diagnosis of external root resorption in replanted permanent teeth. Braz. Oral. Res..

[32] Alqerban A, Jacobs R, Souza PC, Willems G (2009). In-vitro comparison of 2 cone-beam computed tomography systems and panoramic imaging for detecting simulated canine impaction-induced external root resorption in maxillary lateral incisors. Am. J. Orthod. Dentofacial. Orthop..

[33] Neves FS, Vasconcelos TV, Vaz SL, Freitas DQ, Haiter-Neto F (2012). Evaluation of reconstructed images with different voxel sizes of acquisition in the diagnosis of simulated external root resorption using cone beam computed tomography. Int. endod. J..

[34] Neves FS, de Freitas DQ, Campos PS, de Almeida SM, Haiter-Neto F (2012). In vitro comparison of cone beam computed tomography with different voxel sizes for detection of simulated external root resorption. J. Oral. Sci..

[35] Nikneshan S, Valizadeh S, Javanmard A, Alibakhshi L (2016). Effect of voxel size on detection of external root resorption defects using cone beam computed tomography. Iran. J. Radiol..

[36] Safi Y, Ghaedsharaf S, Aziz A, Hosseinpour S, Mortazavi H (2017). Effect of field of view on detection of external root resorption in cone-beam computed tomography. Iran. Endod. J..

[37] Lima TF, Gamba TD, Zaia AA, Soares AD (2016). Evaluation of cone beam computed tomography and periapical radiography in the diagnosis of root resorption. Aust. Dent. J..

[38] Kajan ZD, Taramsari M, Fard NK, Khaksari F, Hamidi FM (2018). The efficacy of metal artifact reduction mode in cone-beam computed tomography images on diagnostic accuracy of root fractures in teeth with intracanal posts. Iran. Endod. J..

[39] Neves FS, Freitas DQ, Campos PS, Ekestubbe A, Lofthag-Hansen S (2014). Evaluation of cone-beam computed tomography in the diagnosis of vertical root fractures: The influence of imaging modes and root canal materials. J. Endod..

